# Expression of concern: Palladium nanoparticles supported on 1,3-dicyclohexylguanidine functionalized mesoporous silica SBA-15 as highly active and reusable catalyst for the Suzuki–Miyaura cross-coupling reaction

**DOI:** 10.1039/d4ra90116a

**Published:** 2024-09-30

**Authors:** Hojat Veisi, Abbas Amini Manesh, Neda Eivazi, Ali Reza Faraji

**Affiliations:** a Department of Chemistry, Payame Noor University (PNU) 19395-4697 Tehran Iran hojatveisi@yahoo.com +98 838 4227463; b Young Researchers & Elite Club, Pharmaceutical Sciences Branch, Islamic Azad University Tehran Iran

## Abstract

Expression of concern for ‘Palladium nanoparticles supported on 1,3-dicyclohexylguanidine functionalized mesoporous silica SBA-15 as highly active and reusable catalyst for the Suzuki–Miyaura cross-coupling reaction’ by Hojat Veisi *et al.*, *RSC Adv.*, 2015, **5**, 20098–20107, https://doi.org/10.1039/C4RA14668A.

*RSC Advances* is publishing this expression of concern in order to alert readers that concerns have been raised over the integrity of the data published in this article. The authors have been contacted but have not responded to requests to provide raw data. An expression of concern will continue to be associated with the article until a conclusive outcome is reached.

Laura Fisher

18th September 2024

Executive Editor, *RSC Advances*

